# Understanding and repurposing CRISPR-mediated alternative splicing

**DOI:** 10.1186/s13059-018-1565-3

**Published:** 2018-11-06

**Authors:** Jordan L. Smith, Haiwei Mou, Wen Xue

**Affiliations:** 1 RNA Therapeutics Institute, University of Massachusetts Medical School, Worcester, MA 01605 USA; 2 Program in Molecular Medicine, Department of Molecular, Cell and Cancer Biology, and Li Weibo Institute for Rare Diseases Research, University of Massachusetts Medical School, Plantation Street, Worcester, MA 01605 USA

## Abstract

Two new studies refine our understanding of CRISPR-associated exon skipping and redefine its utility in engineering alternative splicing.

## Introduction

The simplest iteration of CRISPR/Cas9 (clustered regularly interspaced short palindromic repeats/CRISPR-associated system 9) disrupts gene function by employing one single guide RNA (sgRNA) to localize Cas9 to make double-strand breaks (DSB) at a target genomic site. Once a DSB has been created, the cell begins to repair the DNA through non-homologous end joining (NHEJ), resulting in the insertion or deletion of a small number of nucleotides [1]. Previous surveys of CRISPR/Cas9 off-target effects suggested that the technology is relatively precise, and thus positioned CRISPR/Cas9 as the preferred system for genome editing in the laboratory and potentially in the clinic. Multiple reports now suggest, however, that CRISPR/Cas9 editing results in the unintentional generation of alternatively spliced products, large genomic deletions, translocations and inversions [2–5].

**Fig. 1:**
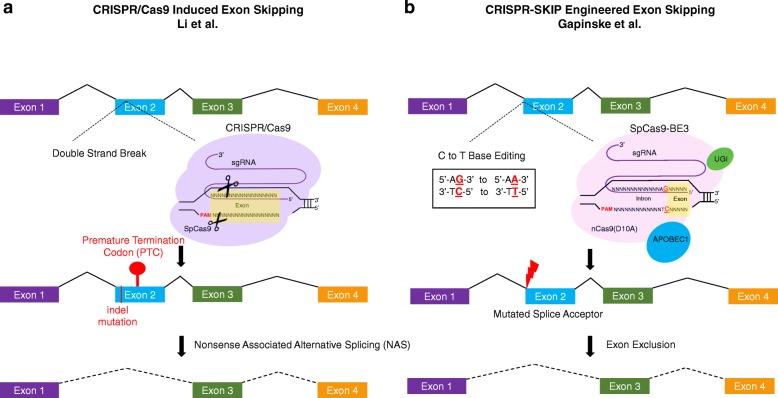
Mechanisms of CRISPR-induced exon skipping. **a** From Li et al. [6], CRISPR/Cas9 induces exon skipping only with the generation of a premature termination codon (*PTC*) in an exon other than exon 1. **b** From Gapinske et al. [7], CRISPR-SKIP repurposes the C > T SpCas9 Base editor, composed of the APOBEC1 cytidine deaminase, the SpCas9-D10A nickase, and the PBS1 uracil glycolase inhibitor (*UGI*), to mutate splice acceptor sites and thus to induce programmable exon skipping. *PAM*, Protospacer adjacent motif; *sgRNA*, single guide RNA

Here, we focus on the alternative splicing induced by CRISPR/Cas9. Several groups have reported alternative splicing following CRISPR/Cas9 editing with a sgRNA [2], but the inciting event for exon skipping remains inconclusive. Two recent publications have begun to both refine our understanding of CRISPR-induced exon skipping and redefine its utility. Specifically, Li and colleagues [6] set out to provide new clarity on how CRISPR-associated indels lead to exon skipping (Fig. 1a). Further, while alternative splicing is frequently considered an undesirable consequence of gene editing, Gapinske et al. [7] show that CRISPR cytosine to thymidine base editors (CBEs) can be repurposed for targeted splicing, adding to the repertoire of tools available for programmable genome editing (Fig. 1b).

## How do CRISPR/Cas9 indels induce exon skipping?

New results from Li et al. [6] suggest that CRISPR/Cas9 induces exon skipping only after the generation of a premature termination codon (PTC). The authors demonstrate that the generation of a PTC following a Cas9-induced DNA break results in nonsense-associated alternative splicing (NAS) and the generation of alternative mRNA products.

The researchers used 22 CRISPR/Cas9 gene edited or CBE rabbit lines. They sorted their mutated rabbit lines by the type of indel: non-frameshift, missense, PTC, and PTC in the first exon. Next, to determine whether the type of indel influences the rate of CRISPR/Cas9-induced exon skipping, they screened their 22 lines by using reverse transcriptase polymerase chain reaction (RT-PCR) to identify exon skipping events. No exon skipping was found in either the non-frameshift rabbit lines or the missense rabbit lines. In the rabbit lines with PTC mutations in exons other than exon 1, however, the researchers detected alternatively spliced mRNA. The results of work by Li et al. [6] therefore suggest that exon skipping occurs only following a PTC mutation, establishing a new rule for the prediction of when exon skipping may occur. Specifically, exon skipping is not dependent on the presence of DNA damage or an indel; rather, a CRISPR indel can only result in exon skipping if it generates a PTC in an exon other than exon 1 (Fig. 1a).

## Purposeful alternative splicing with CRISPR-SKIP

While exon skipping has most often been regarded as an off-target effect that must be mitigated, previous reports have recognized the potential use of CRISPR/Cas9 alternative splicing for disease correction [2]. Targetable exon exclusion strategies have already shown potential therapeutic benefit in many monogenic diseases, including Duchenne’s muscular dystrophy, and Huntington’s disease [8]. Recent work by Gapinske et al. [7] harnesses the unique precision of CBEs to create a new biomedical tool for programmable gene splicing, termed CRISPR-SKIP [7].

Because nearly every intron ends with a guanine, the authors hypothesized that CBEs may be used to disrupt the highly conserved splice acceptor consensus sequence for the exclusion of the following exon. Cystine to thymidine (C > T) CBEs have been shown previously to mutate guanine sites successfully by converting the complementary base, cystine [9, 10]. To test their hypothesis, Gapinske et al. [7] employed a C > T SpCas9 Base editor, composed of the APOBEC1 cytidine deaminase, the SpCas9-D10A nickase, and the PBS1 uracil glycolase inhibitor (Fig. 1b).

For simple detection of exon skipping, Gapinske et al. [7] selected exon 7 of RELA as a test locus because its length, a multiple of three, limits the likelihood that base editing would create a frameshift mutation and trigger nonsense-mediated decay. In conjunction with exon 7 of RELA, the authors also targeted the splice acceptor of exon 5 in PIK3CA. Using deep sequencing, the authors found a base-editing rate of 6.26% G > C in RELA and 26.38% in PI3KCA. These percentages corresponded to an exon skipping rate of 15.46% in RELA and 37.5% in PI3KCA. Surprisingly, at the exon 5 PI3KCA splice acceptor site, the authors also detected G > C (14.66%), G > T (2.58%), and a G > A (10.34%) modifications more than 20-nucleotides outside the CBE target range.

Gapinske et al. [7] also compared the rate of exon skipping generated by CRISPR-SKIP to that of skipping induced by CRISPR/Cas9 following a DSB, as described by Li et al. [6]. With sgRNAs that were not targeted to the splice acceptor, CRISPR/Cas9 induced either an equivalent number of or fewer exon skipping events than CRISPR-SKIP. When the authors used the same sgRNAs targeted to the splice acceptor for both CRISPR-SKIP and CRISPR/Cas9, they found that CRISPR/Cas9 was more effective at inducing exon skipping at three of the five targets, whereas CRISPR-SKIP was more effective at the other two. Further, the authors sought to expand CRISPR-SKIP’s utility by eliminating its dependence on the presence of an NGG protospacer adjacent motif (PAM) 12–17 bp from the target cytidine. They successfully demonstrated that CBE with different Cas9 scaffolds, including SpCas9-VQR-BE3 with NGA PAM and SaCas9-KKH-BE3 with NNNRRT PAM, can induce targeted exon skipping.

Finally, to ease the burden of identifying suitable transcripts for CRISPR-SKIP base editing, Gapinske et al. [7] developed a web-based software tool that allows researchers to identify appropriate sgRNAs for a desired target, incorporates the various CBEs and their efficiency, and generates an off-target score.

## Skipping forward

As CRISPR/Cas9 gene editing accelerates from the bench to the clinic, understanding and perhaps exploiting the unintentional consequences, including exon skipping, translocations, inversion, and deletions, will take center stage. Two recent publications from Li et al. [6] and Gapinske et al. [7] refine our understanding of how CRISPR/Cas9 indels induce exon skipping, and further broaden the CRISPR tool-kit to include programmable exon skipping.

Li et al. [6] sheds new light on how CRISPR/Cas9 gene inactivation inadvertently results in exon skipping. The authors’ finding that only PTC mutations induce exon skipping narrows the hunt for the inciting event of exon skipping, limiting the likelihood that DNA damage or the indel itself have causative roles. These authors suggest that their finding further supports the hypothesis of a ‘nuclear scanning mechanism’ that enables the cell to identify pre-mRNAs with PTCs and shuttles these transcripts through nonsense-associated alternative splicing. We do not yet know how the cell identifies these transcripts, and why the location of the PTC determines whether the transcript undergoes nonsense-mediated decay or nonsense-associated alternative splicing. Further, the role of cis-regulatory elements, specifically exonic splicing silencers, remains elusive. In Li et al.’s study, several missense rabbit lines had disruption of exonic regulatory elements, but only premature PTC lines resulted in exon skipping. Li et al. [6] provides researchers with a new guideline to screen for the presence of alternative mRNA products following CRISPR/Cas9 editing. Specifically, it’s advisable to sequence through your indel, and if it is a predicted PTC, run a RT-PCR to screen for the presence of alternative mRNA products.

Although exon skipping is often viewed as an unintended consequence of CRISPR/Cas9 gene editing, Gapinske et al. [7] harnessed CBEs in CRISPR-SKIP to achieve intentional programmable alternative splicing. CRISPR-SKIP has potential broad utility in both biotechnology and the clinic. Gapinske et al. [7] estimate that 118,089 out of 187,636 inner exons in protein-coding genes are targetable. CRISPR-SKIP could be harnessed as a therapeutic tool to address genetic disease by directing the expression of specific mRNA transcripts. In addition, unlike other exon skipping platforms, CRISPR-SKIP induces stable changes and thus increases the likelihood that a single treatment may be therapeutic for patients. Perhaps the most critical advantage of CRISPR-SKIP over other exon-skipping technologies, including canonical CRISPR/Cas9, is that it does not introduce high-levels of DSBs into the genome, reducing the probabilities of large deletions, translocations and inversions observed with Cas9 [2, 3].

The two articles highlighted here generate further questions around critical gaps in our understanding of how and when splicing occurs in CRISPR-edited systems. For example, Gapinske and colleagues [7] found that they were able to edit approximately 77% of the splice acceptors that were targeted, but only 50% of subsequent exons were skipped. Missed exon skipping may be the result of cryptic splice acceptor sites, incomplete understanding of intron-exon biology, or the need for optimization of base-editing technologies.

Taken together, these two publications provide a critical framework for understanding the mechanism and utility of CRISPR-induced exon skipping.

## Abbreviations

Cas9: CRISPR-associated system 9
CBE: Cytosine to thymidine base editor
CRISPR/Cas9: Clustered regularly interspersed palindromic repeats, Cas9
DSB: Double-strand break
PAM: Protospacer adjacent motif
PTC: Premature termination codon
RT-PCR: Reverse transcriptase polymerase chain reaction
sgRNA: Single guide RNA

## References

[1] Doudna JA, Charpentier E (2014). Genome editing. The new frontier of genome engineering with CRISPR-Cas9. Science.

[2] Mou H, Smith JL, Peng L, Yin H, Moore J, Zhang XO (2017). CRISPR/Cas9-mediated genome editing induces exon skipping by alternative splicing or exon deletion. Genome Biol.

[3] Kosicki M, Tomberg K, Bradley A (2018). Repair of double-strand breaks induced by CRISPR-Cas9 leads to large deletions and complex rearrangements. Nat Biotechnol.

[4] Li Y, Park AI, Mou H, Colpan C, Bizhanova A, Akama-Garren E (2015). A versatile reporter system for CRISPR-mediated chromosomal rearrangements. Genome Biol.

[5] Canver MC, Bauer DE, Dass A, Yien YY, Chung J, Masuda T (2014). Characterization of genomic deletion efficiency mediated by clustered regularly interspaced short palindromic repeats (CRISPR)/Cas9 nuclease system in mammalian cells. J Biol Chem.

[6] Sui T, Song Y, Liu Z, Chen M, Deng J, Xu Y, Lai L, Li Z (2018). CRISPR-induced exon skipping is dependent on premature termination codon mutations. Genome Biol.

[7] Gapinske M, Luu A, Winter J, Woods WS, Kostan KA, Shiva N (2018). CRISPR-SKIP: programmable gene splicing with single base editors. Genome Biol.

[8] Aartsma-Rus A, van Ommen GJ (2007). Antisense-mediated exon skipping: a versatile tool with therapeutic and research applications. RNA.

[9] Gaudelli NM, Komor AC, Rees HA, Packer MS, Badran AH, Bryson DI, Liu DR (2017). Programmable base editing of A*T to G*C in genomic DNA without DNA cleavage. Nature.

[10] Komor AC, Kim YB, Packer MS, Zuris JA, Liu DR (2016). Programmable editing of a target base in genomic DNA without double-stranded DNA cleavage. Nature.

